# Clinical actionability of BRCA2 alterations in uterine leiomyosarcoma: a molecular tumor board case report and a cBioPortal comprehensive analysis

**DOI:** 10.1093/oncolo/oyae082

**Published:** 2024-05-08

**Authors:** Luca Boscolo Bielo, Matteo Repetto, Edoardo Crimini, Carmen Belli, Elisabetta Setola, Gabriella Parma, Nicola Fusco, Massimo Barberis, Elena Guerini Rocco, Antonio Marra, Nicoletta Colombo, Giuseppe Curigliano

**Affiliations:** Division of New Drugs and Early Drug Development for Innovative Therapies, European Institute of Oncology, IRCCS, Milan, Italy; Department of Oncology and Hemato-Oncology, University of Milan, Milan, Italy; Early Drug Development Service, Memorial Sloan Kettering Cancer Center, New York, NY, USA; Division of New Drugs and Early Drug Development for Innovative Therapies, European Institute of Oncology, IRCCS, Milan, Italy; Department of Oncology and Hemato-Oncology, University of Milan, Milan, Italy; Division of New Drugs and Early Drug Development for Innovative Therapies, European Institute of Oncology, IRCCS, Milan, Italy; Melanoma, Sarcoma and Rare Tumors Oncology Department, European Institute of Oncology (IEO) IRCCS, Milan, Italy; Department of Gynecology, European Institute of Oncology (IEO) IRCCS, Milan, Italy; Department of Oncology and Hemato-Oncology, University of Milan, Milan, Italy; Division of Pathology, IEO, European Institute of Oncology IRCCS, Milan, Italy; Division of Pathology, IEO, European Institute of Oncology IRCCS, Milan, Italy; Department of Oncology and Hemato-Oncology, University of Milan, Milan, Italy; Division of Pathology, IEO, European Institute of Oncology IRCCS, Milan, Italy; Division of New Drugs and Early Drug Development for Innovative Therapies, European Institute of Oncology, IRCCS, Milan, Italy; Department of Gynecology, European Institute of Oncology (IEO) IRCCS, Milan, Italy; Department of Medicine and Surgery, University of Milan-Bicocca, Milan, Italy; Division of New Drugs and Early Drug Development for Innovative Therapies, European Institute of Oncology, IRCCS, Milan, Italy; Department of Oncology and Hemato-Oncology, University of Milan, Milan, Italy

**Keywords:** uterine leiomyosarcoma, niraparib, next-generation sequencing, BRCA2 homozygous deletion

## Abstract

**Background:**

Uterine leiomyosarcoma (uLMS) represents one of the most common sarcoma histotypes, demonstrating an overall dismal prognosis. Previous studies reported uLMS to carry recurrent somatic *BRCA2* homozygous deletions, related to significant clinical benefits from the use of PARP inhibitors.

**Methods:**

To investigate the prevalence in uLMS of genomic alterations (^alt^) in *BRCA2* and other homologous recombination (HR) and DNA damage response (DDR) genes, cBioPortal was accessed and data were retrieved from studies including pan-sarcoma histologies. HR-/DDR-genes included *BRCA1*, *BRCA2*, *ATM*, *BARD1*, *BRIP1*, *CHEK1*, *CHEK2*, *FANCA*, *FANCB*, *FANCC*, *FANCD2*, *FANCE*, *FANCF*, *FANCG*, *FANCI*, *FANCL*, *FANCM*, *NBN*, *PALB2*, *RAD51C*, *RAD51D*, *RAD50*, and *ATR*. Only oncogenic/likely oncogenic alterations were included according to OncoKB.

**Clinical Report and Results:**

We reported a clinical case of a patient affected by a highly pretreated uLMS discussed at the European Institute of Oncology Molecular Tumor Board. A targeted next-generation sequencing panel demonstrated a somatic *BRCA2* homozygous deletion (homDel). Upon access to Niraparib, a remarkable response of 15 months was observed before experiencing disease progression. In the genomic query, among 2393 cases, uLMS (*n* = 193) displayed 9 of all 31 *BRCA2*^*alt*^ observed, representing the only sarcoma histotype showing an enrichment in *BRCA2*^*alt*^ (4.66%; *q* < 0.001). All of 9 *BRCA2*^*alt*^ were represented by homDel, which related to a high fraction of genome altered.

**Conclusion:**

uLMS displays a significant frequency of somatic *BRCA2*^*alt*^ homDel. Considering their dismal prognosis, further investigation is warranted to test the use of PARPi in uLMS, and particularly in the setting of *BRCA1/2* alterations.

## Teaser

This Molecular Tumor Board report describes a remarkably response achieved with the use of Niraparib in a patients affected by uterine leiomyosarcoma carrying a somatic biallelic BRCA2 alteration. In the accompanying genomic analysis, an enrichment of BRCA2 somatic alterations were observed among uterine leiomyosarcoma, confirming their prevalence and potential clinical actionability.

**Key points**

- Prolonged response to a poly (ADP-ribose) polymerase inhibitor was observed in a patient affected by highly pretreated uterine leiomyosarcoma (uLMS) carrying a somatic, homozygous BRCA2 deletion.
- BRCA2 alterations are enriched in uLMS compared to other sarcoma histotypes.
- Homozygous deletions account for most BRCA2 alterations in uLMS.
- BRCA1/2 homozygous deletions yield high genomic instability.
- Further investigation is mandatory for the use of PARPi in uLMS carrying BRCA2 alterations.

## Case presentation

On December 2011, a 60-year-old female was diagnosed with a uterine leiomyosarcoma (uLMS) after undergoing a bilateral hysteroannessiectomy. Following surgery, 4 cycles of adjuvant therapy of gemcitabine with docetaxel were administered until May 2012.

**Figure 1.**
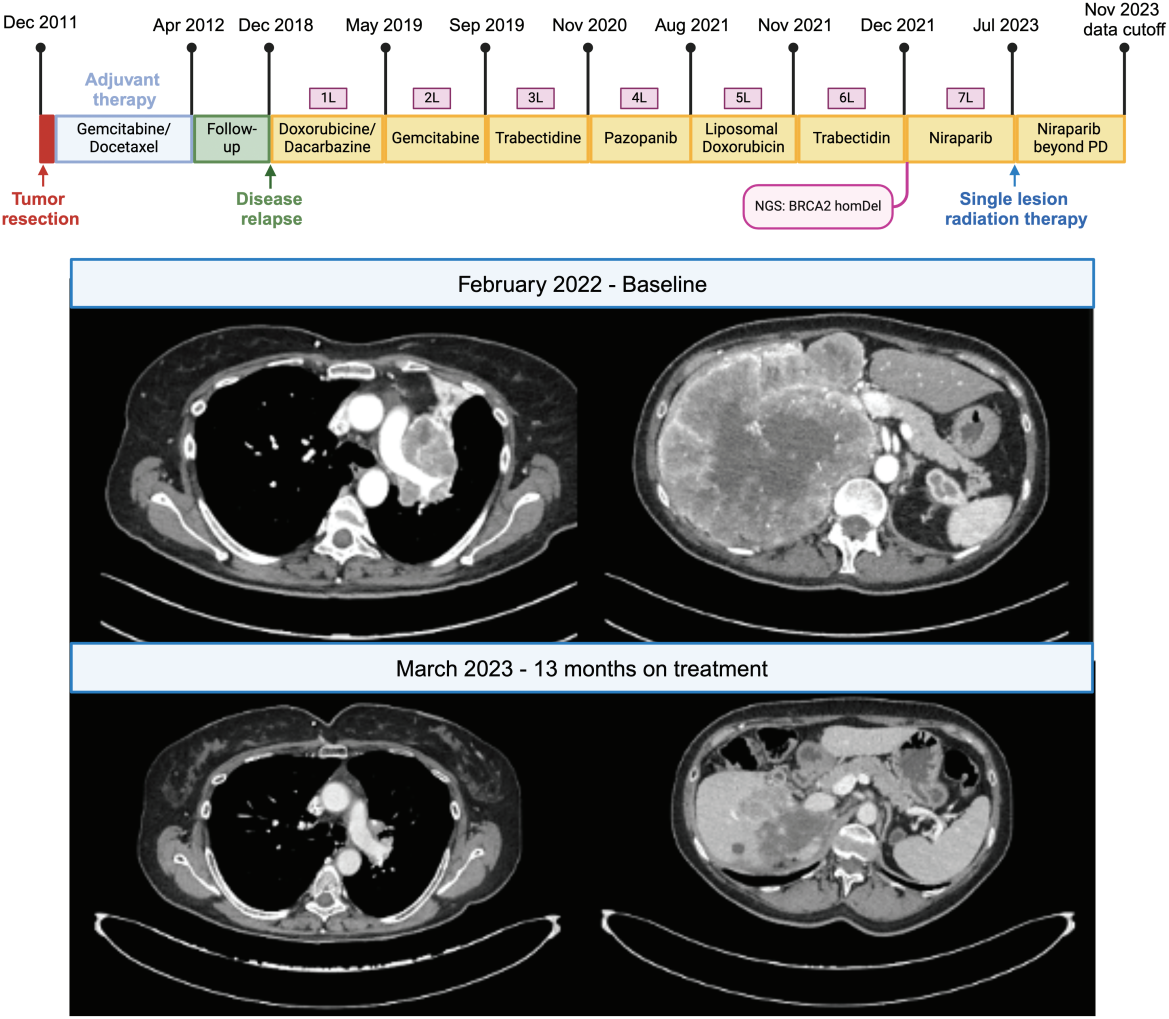
Patient oncological history and target lesions assessment.

After a negative follow-up, a relapse was observed with disease recurrence in the lungs and adrenal glands on December 2018. Subsequent treatments included doxorubicin plus dacarbazine; gemcitabine; trabectedin; pazopanib; liposomal doxorubicin; and trabectedin rechallenge, ultimately showing progressive disease on December 2021 (Figure 1).

On January 25, 2022, with no additional standard treatments available, patients received a comprehensive genomic profiling with next-generation sequencing (NGS; see Methods), whose report was referred to the European Institute of Oncology (IEO) Molecular Tumor Board (MTB). NGS was performed on the primary tumor tissue dated to the time of surgery, with genomic signatures and alterations (^alt^) reported in Table 1. Of note, a BRCA2 deletion was found, whose somatic origin was confirmed by a negative germline test. Considering the rationale for *BRCA2* actionability and the absence of molecular alterations suggestive of primary resistance to poly (ADP-ribose) polymerase inhibitors (PARPi), an indication to the off-label use of PARPi was recommended by the MTB, which patient received from February 2022. Targeted treatment with Niraparib resulted tolerable and showed a durable radiological partial response which lasted until June 2023 (Figure 1), when disease progression occurred in a single liver lesion. Subsequent radiation therapy was administered to the progressing lesion on July 2023, with patient still receiving Niraparib 4 months after local radiation therapy.

**Table 1.** Alterations found in the targeted NGS panel. VUS, variants of uncertain significance.

Genes	Alteration			Annotation
	Protein	Coding	TRANSCRIPT ID	
BRCA2	\	Loss	\	Pathogenic
ATRX	\	c.5273-1G>A	NM_000489	Pathogenic
C17orf39	\	Amplification	\	Pathogenic
NCOR2	\	6980-100_7023del144	NM_006312	Pathogenic
RB1	\	Loss exons 18-27		Pathogenic
TP53	p.P278S	c.832C>T	NM_000546	Pathogenic
EPHA7	\	Loss	\	VUS
ERBB4	p.N465K	c.1395C>A	NM_005235.3	VUS
FLCN	\	Amplification	\	VUS
FLT4	p.R1070H	\	\	VUS
MAP2K4		Amplification		VUS
MLL2	p.L2973P and p.P692T	\	\	VUS
SPEN	p.S2841G	\	\	VUS

## Methods

### Next-generation sequencing platform and MTB at the European Institute of Oncology

In the presented case, blood-based FoundationOneHEME^1^ was used for genomic analysis. Multiplex Ligation-dependent Probe Amplification (MLPA)^2^ was performed on peripheral blood for germline BRCA2 testing.

IEOs MTB includes oncologists, molecular pathologists, molecular biologists, geneticists, radiotherapists, and pharmacologists, as previously reported.^3^

Patient discussed at the MTB whose case is reported provided informed consent. The present work was approved by the IEO internal review board and was conducted in accordance with the principles of the Declaration of Helsinki and with the principles of good clinical practice.

### cBioportal genomic analysis to investigate the prevalence of alterations affecting homologous recombination/DNA damage response genes among sarcoma histologies

In our genomic analysis, our primary aim was to investigate the prevalence of *BRCA2* and other homologous recombination (HR)/DNA damage response (DDR) alterations in uLMS as compared to other sarcoma histotypes. cBioPortal^4,5^ was queried for publicly available genomic and clinical data using the cBioPortalR package.^6^ Data were extracted from studies including uLMS, filtered for patients duplicated across selected repositories. HR-/DDR-genes selected in the genomic query and subsequent analysis included *BRCA1*, *BRCA2*, *ATM*, *BARD1*, *BRIP1*, *CHEK1*, *CHEK2*, *FANCA*, *FANCB*, *FANCC*, *FANCD2*, *FANCE*, *FANCF*, *FANCG*, *FANCI*, *FANCL*, *FANCM*, *PALB2*, *RAD51C*, *RAD51D*, *RAD50*, *NBN*, and *ATR*. Oncogenic and likely oncogenic alterations were included in the analysis according to OncoKB.^7^.

### Statistical analysis

In the genomic analysis, categorical variables were reported as absolute number and proportion, and continuous variables as median and interquartile range. Categorical variables were compared using the Fisher’s exact test or chi-squared test, as appropriate. Bartlett test and Shapiro-Wilk test were used to assess variances and normal distributions, respectively. Non parametrical test for continuous variable included the Wilcox test and Kruskal-Wallis test. Dunn’s test was used for multiple pairwise comparisons after a significant Kruskal-Wallis test. False discovery rate was used for multiple comparisons. All tests were performed using a 2-sided significance level of <.05. Statistical analysis was performed using R Software version 4.3.2.^8^

## Genomic analysis

**Figure 2.**
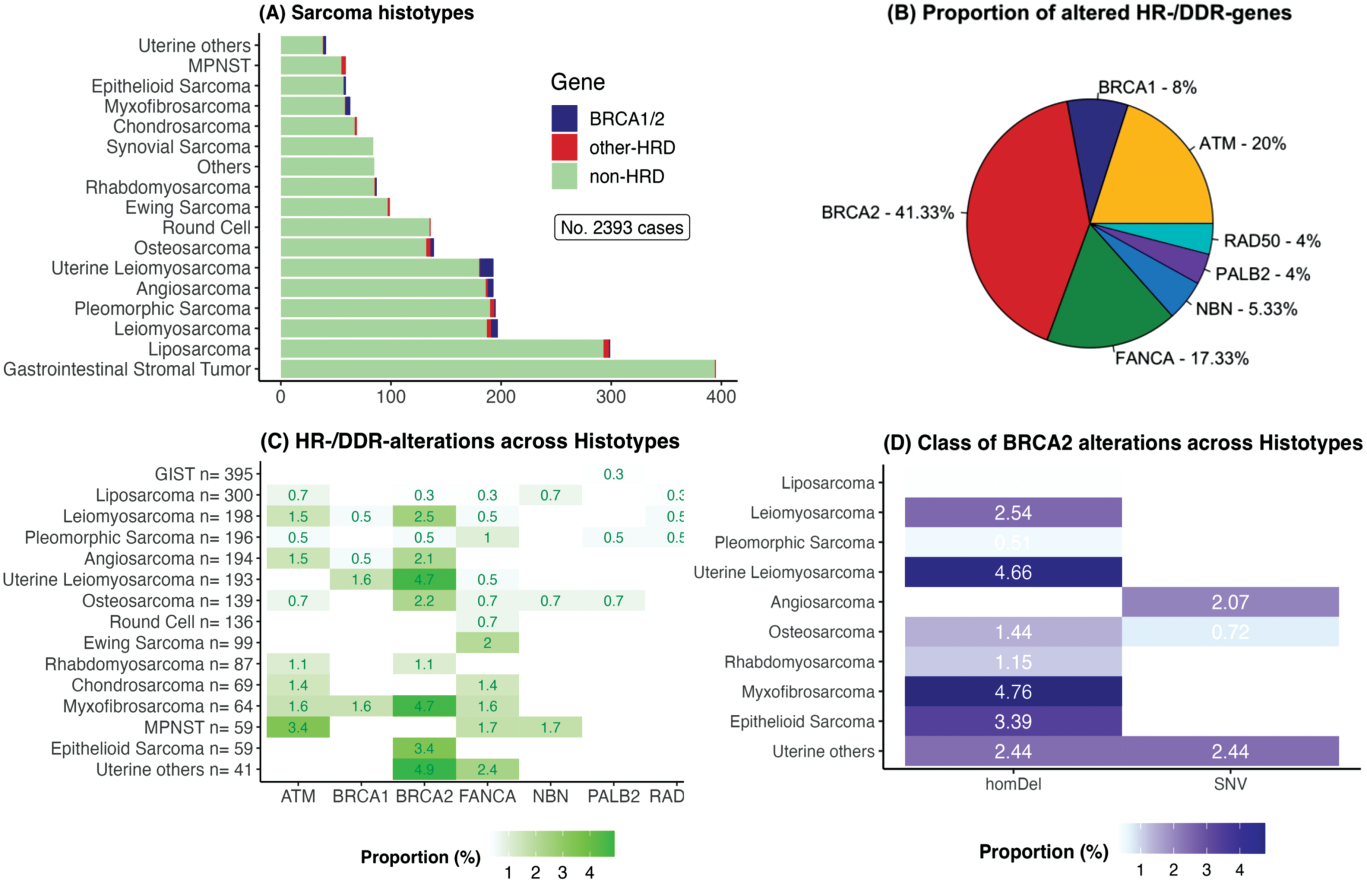
Distribution of genes and classes of HR-/DDR-alterations across sarcoma histotypes. homDel, homozygous deletion; MPNST, malignant peripheral nerve sheath tumor; NO, number; SNV, single-nucleotide variant.

**Figure 3.**
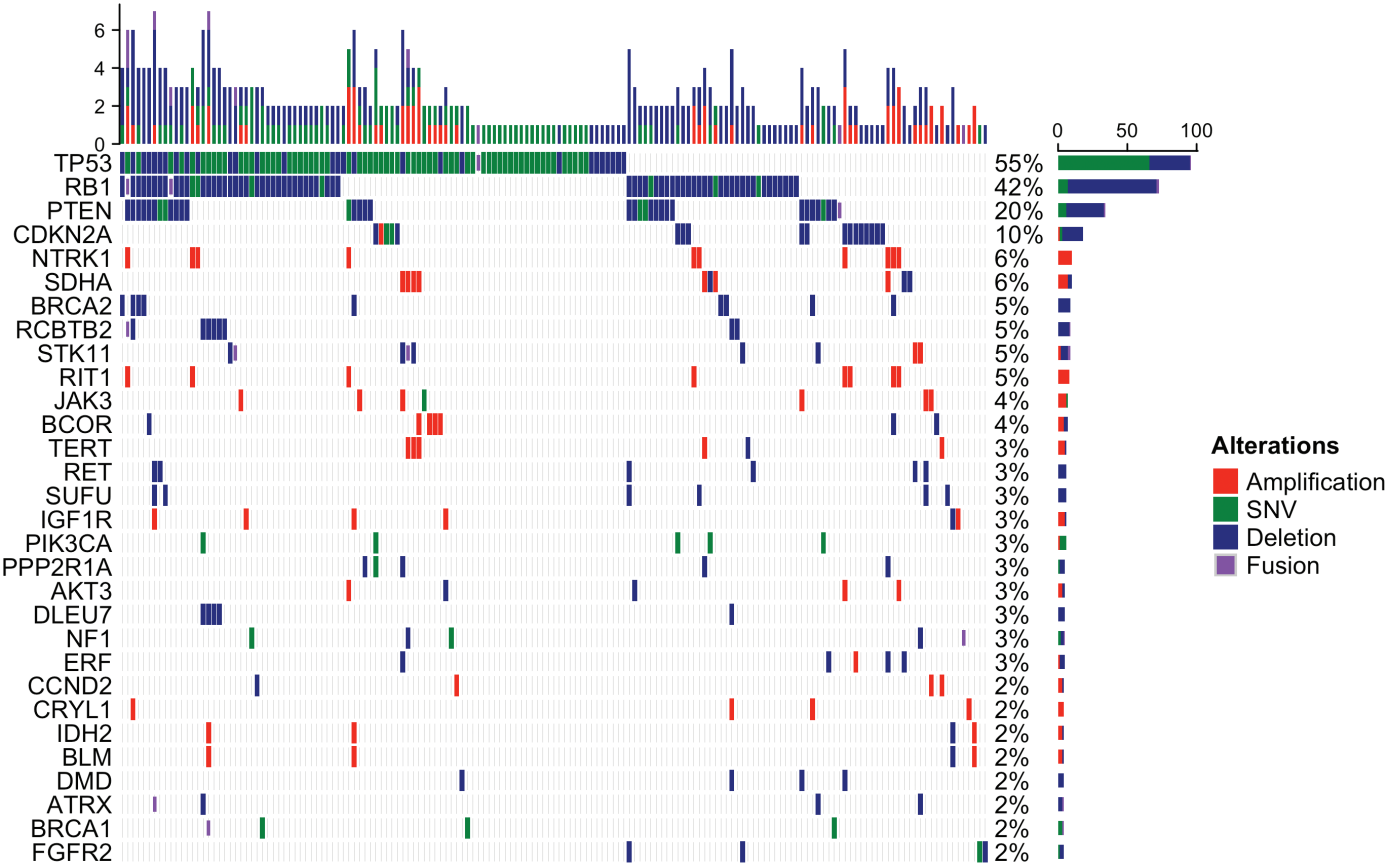
Oncoprint of genomic alterations in uterine leiomyosarcoma. SNV, single-nucleotide variant.

A total of 2393 patients affected by sarcoma were retrieved, among which uLMS represented the sixth most common histotype (*n* = 193, 8.07%; Figure 2A). Across histotypes, 75 HR-/DDR-gene^alt^ in 70 cases were observed, with *BRCA2* showing the highest frequency (31/75, 41.33%; Figure 2B). LMS accounted for 48.65% (18 of 37) of all *BRCA1/2*^*alt*^, with 14 of 18 (77.78%) represented by *BRCA2*^*alt*^ (Figures 2C and 3). uLMS showed a higher proportion of *BRCA1/2*^*alt*^ compared to nonuterine Leiomyosarcoma (non-uLMS; 6.21% vs. 3.04%, *P* = .21). Across histotypes, both uLMS (6.21%; *q* < 0.01) and myxofibrosarcoma (6.35%; *q* = 0.01) showed an enrichment of *BRCA1/2*^*alt*^, while only uLMS showed enrichment in *BRCA2*^*alt*^ when excluding *BRCA1*^*alt*^ (uLMS 4.66%, *q* < 0.001; Figure 2D).

*BRCA1/2* ^*alt*^ classes were unevenly distributed, with homDel representing most of *BRCA1/2*^*alt*^ (70.27%, 26 of 37, *P* < .001) and *BRCA2*^*alt*^ (80.65%, 25 of 31, *P* < .001). Of note, all 9 *BRCA2*^*alt*^ in uLMS consisted in homDel.

Tumors carrying *BRCA1/2*^*alt*^ showed higher fraction genome altered (FGA) compared to HR-/DDR-wild-type tumors (0.32 [interquartile range, IQR, 0.21-0.52] vs. 0.16 [IQR 0.04-0.34]; *P* < .01) but not compared to non-BRCA1/2 HR-/DDR-alterations (vs. 0.29 [IQR 0.09-0.46]; *P* = .33). HomDel in *BRCA2* yielded higher FGA compared to *BRCA2* single-nucleotide variants (0.409 [IQR 0.29-0.56] vs. 0.014 [IQR0.005-0.128], *P* = .003).

## Discussion

In the presented case, we reported a long-lasting response to PARPi in a patient affected by a highly pretreated uLMS carrying a somatic BRCA2 homozygous deletion.

Our observation is consistent with previous findings. In a case series of Seligson and colleagues,^9^ prolonged responses to olaparib were observed among 4 highly pretreated uLMS, with 3 of them carrying somatic BRCA2 deletions and 1 showing a truncating BRCA2 alteration. Similarly, prolonged responses to PARPi were observed among 4 uLMS demonstrating BRCA2 homDel in the study of Hensley et al,^10^ with other similar studies corroborating the remarkable efficacy of PARPi in this setting of disease.^11,12^

Several trials testing the use of PARPi in sarcomas are currently ongoing. Preliminary results of the phase II TOMAS2 trial did not demonstrate benefits from the addition of olaparib to trabectedin among 130 patients affected by sarcomas, with a 6-month PFS rate of 32% (95% CI 22%-46%) as compared to 28% (95% CI 19%-42%) in the control group (*P* = 0.122).^13^ Of note, despite preclinical evidence of trabectedin to enhance the activity of olaparib irrespective of HR-/DDR-alterations,^14^ no biomarker was considered for patients inclusion in the study, which could have led to a low number of cases ultimately showcasing predictive biomarkers of PARPi efficacy. Indeed, as we observed in our analysis, alterations in HR-/DDR-genes occur infrequently among sarcomas, not suggesting the indiscriminate use of PARPi in sarcomas, either alone or with chemotherapy, might yield clinical benefits and cost-effective treatment strategies.

In our analysis, across sarcoma histotypes, *BRCA2* was found to be the most commonly altered HR-/DDR-gene. Noteworthy, of all *BRCA2*^*alt*^ observed among histotypes, 29.03% (9 of 31) occurred in uLMS, found in 4.66% of cases, in line with previous reports.^10,15^ Specifically, in uLMS *BRCA2*^*alt*^ represented 60% (9 of 15) of all HR-/DDR-genes defects. Therefore, HR-pathway alterations in uLMS are predominantly driven by *BRCA2*^*alt*^, which occur with a relevant frequency.

Of note, we observed all *BRCA2*^*alt*^ in uLMS being represented by homDel, involving the structural deletion of both alleles. Biallelic alterations in HR-/DDR-genes are increasingly recognized as a genomic biomarker of HRD and PARPi sensitivity, and particularly for homozygous deletions preventing the occurrence of BRCA1/2 reversal alterations.^16–20^ Albeit germline HR-/DDR-alterations generally relate to a higher proportion of biallelic compared to monoallelic alterations, in a pancancer analysis uLMS exhibited the highest frequency of BRCA2 somatic biallelic alterations.^21^ In the same study, all BRCA2 alterations consisted of homDel of somatic origin,^21^ as we observed in our case report and genomic analysis. Accordingly, in uLMS, biallelic *BRCA2*^*alt*^, mainly consisting in structural variants, occurs at relevant biallelic rates despite of their somatic origin.

Besides alterations in HR-/DDR-genes, previous studies reported 25%-30% of uLMS to carry a COSMIC mutational signature 3, which acts as a genomic surrogate of HRD and PARPi responsiveness.^12^ Regardless, few data are available to relate signatures of HRD with PARPi sensibility in uLMS. In the study of Dall and colleagues, all 13 of 58 uLMS subjected to whole-genomic sequencing displayed a COSMIC mutational signature 3, with one patient receiving PARPi demonstrating a minor response at 4 months before interrupting the treatment due to toxicity.^12^ In a single-arm, phase II trial evaluating the combination of olaparib plus temozolomide in 22 uLMS, despite no *BRCA1*/*2*^*alt*^ were observed, 50% of cases demonstrated HRD by *RAD51* assay,^22^ which correlated with prolonged PFS from olaparib plus temozolomide (PFS 11.2 vs. 5.4 months; *P* = .05).^23^ Additionally, in the TOMAS2 trial, while no benefits were observed from the addition of olaparib to trabectedin in the prespecified subgroup analysis of LMS (*n* = 130), in an exploratory analysis a higher benefit was observed from olaparib among LMS showing HRD, defined as a level of Genomic Instability Score above the median (6-month PFS rate of 46 [95% CI 26%-83%] vs. 20% [95% CI 6%-69%], *P* = .053).^13^ Altogether, these data suggest a larger cohort of patients affected by uLMS, and possibly non-uLMS, could potentially benefit from the use of PARPi. Accordingly, substantial rationale exists for the design of clinical trials leveraging on HRD-related biomarkers for testing PARPi in uLMS in a biomarker-driven strategy.

It must be noted that our work presents some limitations. Our retrospective, exploratory analysis included a limited number of patients showing HR-/DDR-genes alterations, and thus our results should be interpreted with caution. In addition, in the genomic analysis, we could not discriminate between somatic and germline genomic alterations. Moreover, we could not distinguish allele-specific status of HR-/DDR-genes alterations, as no access to raw sequencing data was available. Lastly, our retrieved data lacked information about anti-neoplastic treatments and clinical follow-up for patients included in the genomic query.

## Conclusion

Our presented case corroborates similar findings reporting the remarkable efficacy of PARP inhibitors in the context of somatic *BRCA2* homozygous deletions in uLMS. In addition, our genomic analysis underscores the prevalence of *BRCA2* alterations in uLMS, emphasizing the importance of genomic profiling to detect the subgroup of patients affected by uLMS which might potentially benefit from the use of PARPi. Accordingly, further research to test the use of PARPi in uLMS is demanded. Furthermore, our findings highlight the infrequent occurrence of HR-/DDR-gene alterations in sarcomas, advocating for a refined patient selection strategy in clinical trials testing the use of PARPi across sarcoma histotypes.

## Data Availability

The data underlying this article will be shared on reasonable request to the corresponding author.
